# Deep-Learning-Based detection of recreational vessels in an estuarine soundscape in the May River, South Carolina, USA

**DOI:** 10.1371/journal.pone.0302497

**Published:** 2024-07-08

**Authors:** Yiming Ji, Alyssa D. Marian, Eric W. Montie

**Affiliations:** 1 Department of Information Technology, Georgia Southern University, Statesboro, GA, United States of America; 2 Department of Natural Sciences, University of South Carolina Beaufort, Bluffton, South Carolina, United States of America; Whale Wave Technology Inc, CHINA

## Abstract

This paper presents a deep-learning-based method to detect recreational vessels. The method takes advantage of existing underwater acoustic measurements from an Estuarine Soundscape Observatory Network based in the estuaries of South Carolina (SC), USA. The detection method is a two-step searching method, called Deep Scanning (DS), which includes a time-domain energy analysis and a frequency-domain spectrum analysis. In the time domain, acoustic signals with higher energy, measured by sound pressure level (SPL), are labeled for the potential existence of moving vessels. In the frequency domain, the labeled acoustic signals are examined against a predefined training dataset using a neural network. This research builds training data using diverse vessel sound features obtained from real measurements, with a duration between 5.0 seconds and 7.5 seconds and a frequency between 800 Hz to 10,000 Hz. The proposed method was then evaluated using all acoustic data in the years 2017, 2018, and 2021, respectively; a total of approximately 171,262 2-minute.wav files at three deployed locations in May River, SC. The DS detections were compared to human-observed detections for each audio file and results showed the method was able to classify the existence of vessels, with an average accuracy of around 99.0%.

## 1. Introduction

In 2021, there were about 11.96 million registered recreational vessels in the United States, up from 11.84 million in the previous year. However, as Fields [1] and many other researchers indicate, recreational vessels can have environmental consequences. First, the fueling of boats involves spilling gas, diesel fuel, and other toxicants into waterways. Boating also generates noise, pollution (waste, cleaning materials, and even sewage), and other ecosystem hazards that can negatively impact wildlife [2–12]. Moreover, boating is not a risk-free activity for marine life. A vessel can cause injuries and mortality, generate acoustic masking, and change marine habitats. Researchers have reported that boat activities could directly impact diamondback terrapins [4], beluga whales [5–7], bottlenose dolphins [8–10], fish species, including toadfish [11, 12], black drum [11, 13], as well as silver perch, spotted seatrout, and red drum [11], reef ecosystems [2], seagrass coverage [14], and marine biodiversity [15].

Many tools, including accelerometer sensors [16, 17], high-definition cameras [18, 19], radars [20, 21], and wireless sensor networks [16, 21] have been widely used to track vessels. Due to the advancement of recording technology, researchers have also collected underwater sounds at very short time intervals (e.g., continuously, 20-min, or 60-min) and over long-term scales (i.e., years and decades). These data provide a unique insight into the behaviors of marine life and human activities including recreational boating. Consequently, soundscape ecology is evolving into a scientific discipline that investigates the biological (bio-phony), geophysical (geophony), and anthropogenic (anthrophony) sounds that are produced in marine ecosystems [22, 23].

Since soundscape studies utilize passive acoustic recorders that generate a plethora of sound files, research groups have developed automatic detection methods. An early “(adaptive) median constant false alarm rate (AMCFAR) and multi-frame post-detection integration” algorithm in 2004–2005 [24, 25] combined time and frequency domain signal features for vessel detections. Later, Sorensen et al. 2010 [26] developed a wavelet detection algorithm to capture spectrogram and harmonic signatures of boats for detection purposes. Bruno et al. 2011 [20] combined satellite imaging and high-frequency radar systems to achieve both underwater and surface target detection, classification, and tracking. Stevens Institute of Technology reported a Stevens passive acoustic detection system (SPADES) in 2013 to detect both surface and underwater objects using passive acoustic sensors [27]. The NATO-STO Centre for Maritime Research and Experimentation reported, in 2015, the use of a tridimensional volumetric acoustic array on a mobile underwater platform, i.e., an underwater glider, to monitor marine traffic in defined sea areas [28]. More recently, passive sonar [29], acoustic tag [30], and boat engine parameters (such as shaft and engine rate, number of propellers and blades, and engine firing rate) [31] have also been utilized to detect marine vessels. Convolutional neural networks (CNN) [29, 32] and hidden Markov models (HMMs) [33] have been applied, and Frequency Amplitude Variation (FAV) signature [34] has also been investigated in the detection process. Most recently, Wilson [19], in 2022, used a CNN method to count boats in images and further analyzed the relationship between the soundscape and the number of boats. Research results demonstrated potential applications for boat monitoring at remote sites.

Researchers have also taken advantage of the Automatic Identification System (AIS), where data from ship transceivers and marine radar are used for marine vessel traffic services. Cortese et al. [21] presented a multi-sensor surveillance testbed in Plum Island, NY in 2016. Various tools, including radars, cameras, geophones, and underwater passive acoustic sensors, are connected to a Command-and-Control Center via a Wi-Fi network for the detection of both surface and underwater intruders [21]. Various targets, including jet skis, small boats, kayaks, and divers, as well as commercial ships (with AIS receivers) and long-range surface vessels (by radar), were considered in the study. Alvaro et al. 2021 also used AIS for ship estimation, and two methods [35], including k-nearest neighbors (KNN) and logistic regression models, were applied to process passive underwater acoustic recordings. The KNN method achieved a 98.04% accuracy in estimation. Most recently, Spadon et al. 2022 [36] constructed a model to process AIS message transmission behavior through neural networks, including RNN (Recurrent Neural Networks), GRU (Gated Recurrent Unit), and LSTM (Long-Short Memory) networks for forecasting upcoming AIS messages from vessels; and thus, it would be able to track vessels’ trajectories. AIS messages are transmitted over radio or satellite at ideal periodic time intervals but vary irregularly over time, and the purpose is to track vessels’ trajectories [36].

These studies are limited in their geographical scope (e.g. canal [32], shallow water [17] [29], or other controlled conditions [21, 26, 27, 37, 38]) and duration (e.g. days [13, 24, 32, 33], during the summer [5, 7, 10], or only a few months [2, 15, 39]). For example, the research in [32] used a sound recorder with a sampling frequency of 24 kHz to collect data in one day in a canal in Tokyo. Also, research in [29] collected data in a shallow water environment over two days. Moreover, while machine learning methods have been applied to track vessels and boats, research is still limited. Research [32] applied both CNN networks and an LSTM-RNN method and further developed a gated recurrent unit (GRU) enhanced RNN (GRU-RNN). The research reported a 92.5%~95.5% estimation accuracy for these three methods. Research [29] applied CNN for both the detection of vessels and the estimation of the distance between the vessel and the hydrophone. Research [36] applied similar methods (CNN, LSTM-RNN, and GRU-RNN) as [32], however, it used AIS messages for tracking vessels’ trajectories. Research [19] used CNN to monitor boats without the assistance of AIS, however, higher-resolution cameras are needed to get quality timelapse images of moving objects. Thus, an efficient detection method for vessel noise that works in biologically rich, sound environments (e.g., estuarine soundscapes) would be valuable.

This research takes advantage of existing passive acoustic recorders deployed in the May River, South Carolina (SC), USA that partly comprise The Estuarine Soundscape Observatory Network in the Southeast (ESONS). This network has been collecting underwater sound data since 2013. Developing automatic detectors to identify vessels among a soundscape dataset is typically challenging in estuaries because these ecosystems are louder and more acoustically rich than pelagic ecosystems [40–46]. Thus, the May River estuary is an interesting model system, especially because of the recent population boom in Beaufort County, SC. The human population in Beaufort County, SC has grown by about 14.9% between 2012 and 2022 (U.S. Census Bureau). According to the 2021 Beaufort County Atlas [47], registered boats in the county increased by 23% from 2007 to 2014. The goal of this research project was not to design or evaluate a new deep-learning algorithm, instead, the research aims were to develop an automated approach, using existing deep-learning tools, to detect vessel activity from acoustic files collected from passive acoustic recorders. The research first built a set of boat signal features that were manually analyzed from sound files at different locations. The research then proposed a two-step searching method, called Deep Scanning (DS) that analyzes both time-domain signal energy and frequency-domain spectrum profile for detection purposes. The method was evaluated using recordings in the years 2017, 2018, and 2021, about 171,262 audio files, at three locations in May River, and then compared to human-derived vessel detections.

## 2. Methods

### 2.1 Study site

**Fig 1:**
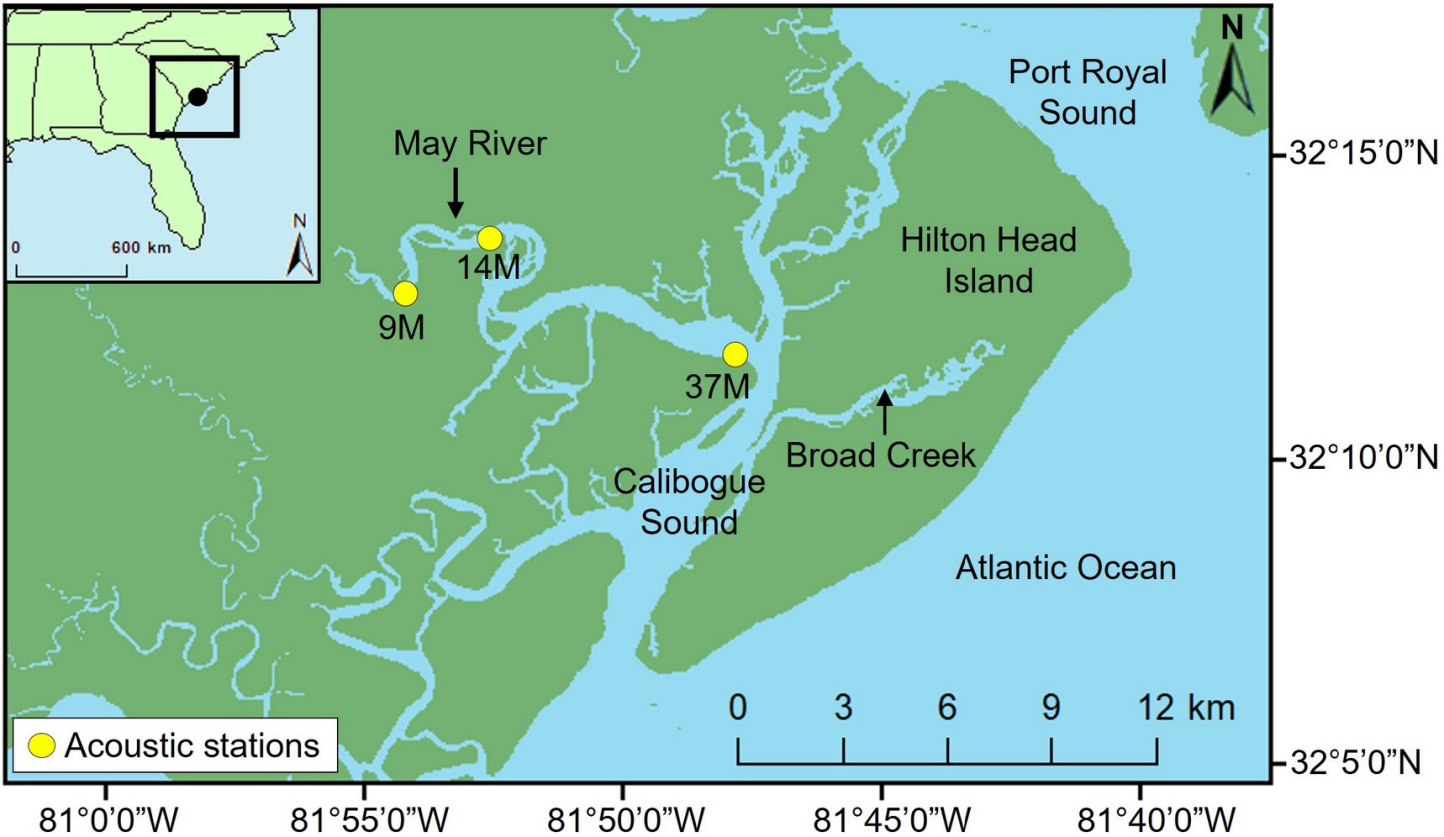
Map of three passive acoustic monitoring stations in the May River estuary, SC that were deployed from 2013 to present. (Inset) The May River estuary (black circle) is in reference to the east coast of the United States. In the figure, 9M, 14M, and 37M are the locations of passive acoustic recorders locations, where 9M was located near the source, 14M was in the middle but also close to the source, and 37M was located at the mouth of the tidal river, near the intra-coastal waterway.

This study takes place in the May River (32° 12’ 49” N, 80° 52’ 23” W), which is a 22 km long estuary located in Beaufort County, SC (Fig 1). May River water depth ranges from 3 to 7 meters at the source (or near 9M) and from 4 to 18 meters at the mouse (near 37M). The river is about 0.5 miles wide at the mouth but becomes narrow when it gets to the source.

This study was conducted under the National Oceanic and Atmospheric Administration’s (NOAA) Policy and Procedures for Compliance with the National Environmental Policy Act and Related Authorities (NOAA Administrative Order 216-6A and Companion Manual for NAO 216-6A), which specifies the determination of the deployment, installation, annual routine operational and maintenance activities conducted by SECOORA awards (numbers NA16NOS0120028 and NA21NOS0120097).

### 2.2 Data collection

**Fig 2:**
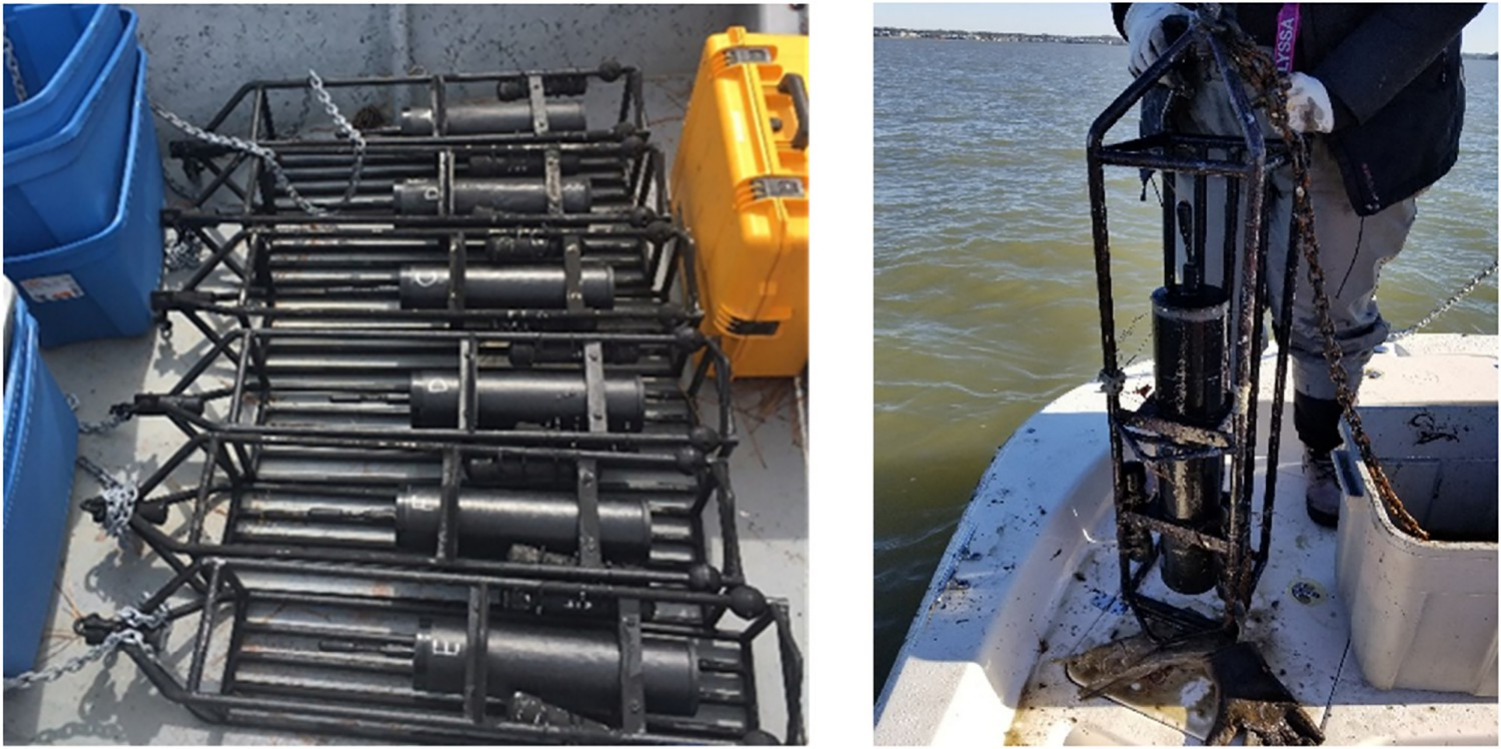
Example photos of instrument frames with passive acoustic recorders as part of the Estuarine Soundscape Observatory Network in the Southeast (ESONS). The left figure shows six recorders before deployment and the right figure shows a platform after deployment.

Each mooring platform consists of a passive acoustic recorder (DSG-Oceans, Loggerhead Instruments), a water level logger (HOBO 100-Foot Depth Water Level Data Logger U20-001-02-Ti, Onset Computer Corporation), and a temperature logger (HOBO Water Temperature Pro v2 U22-001, Onset Computer Corporation) attached to a custom-built instrument frame (Mooring Systems Inc.) (Fig 2). DSG Ocean recorders were equipped with a hydrophone (High Tech) with a sensitivity of -186 dBV μPa-1 and a gain of 20 dB. Recorders were powered with 24 D-cell alkaline batteries and scheduled to record underwater sound for 2 minutes every 20 minutes (from 2013 to 2019) or two minutes every hour (2020 to present) at a sample rate of 80 kHz. Recorders were serviced every 3 months and all sound files were saved on an SD card as a DSG file, which was then downloaded and converted to a.wav file after each deployment.

### 2.3 Vessel signal categorization

**Fig 3:**
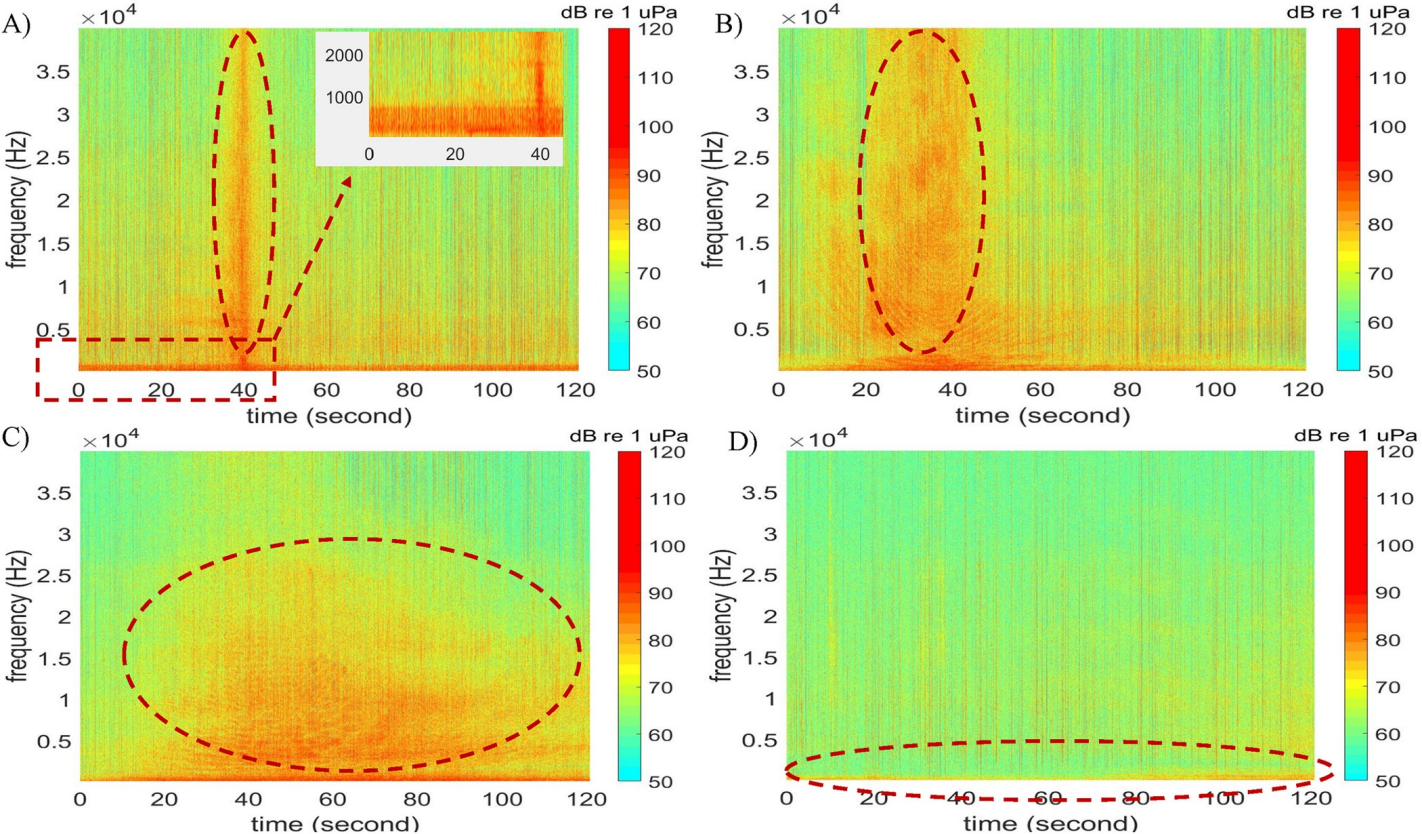
Boat signal patterns identified in spectrogram in the May River estuary from 2017 to 2021. A) Narrow burst broadband, B) wide burst broadband, C) variable broadband, and D) low-frequency signal. Panel A inset highlights details of fish activities at low frequencies (0~800 Hz).

Observers manually reviewed sound files collected every 20 min or every hour using Adobe Audition CS5.5 software (Adobe, Inc.) and identified the absence or presence of vessels. Using this dataset, three boat signal patterns were identified: i) burst broadband (BB), ii) variable broadband (VB), and iii) low-frequency (LF) sounds [11]. The categorization was based solely on the acoustic characteristics observed in spectrograms. The burst broadband signal spans most of the frequency range, both at higher and lower frequency ranges (up to 40 kHz) and appears as a burst. A burst broadband signal typically originates from a vessel traveling by a recorder at a fast speed. Burst broadband signals are further classified into two subtypes according to time duration, which include Burst Narrow (which spans a few seconds as observed in Fig 3A) and Burst Wide (which usually has a longer duration than Burst Narrow as observed in Fig 3B). According to a survey of a dataset measured at station 37M from April to July 2018 (37M_1084_072618, a total of 6815 files, see Section 3, Table 2), the mean Burst Narrow duration was 5.9 ± 2.1 seconds. In comparison, the mean Burst Wide duration was 15.9 ± 6.6 seconds.

The variable broadband signal comes at a low or moderate-speed boat passing the recorder. Variable broadband covers low and moderate frequencies, and it is typically below 25 kHz (Fig 3C). Different from burst broadband, the variable broadband signal generally spans a much longer duration (in minutes). Low-frequency signal comes from boats idling or traveling at a very slow speed. As a result, it usually appears at a lower frequency (below 1.0 kHz) and spans a longer duration (Fig 3C).

### 2.4 Detection method

**Fig 4:**
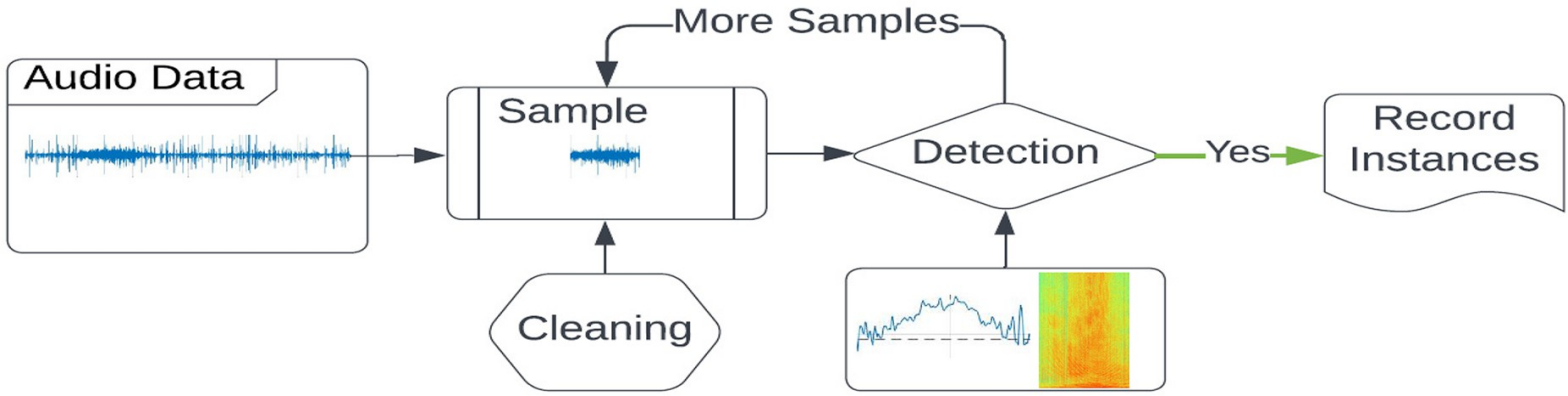
Deep scanning process.

The proposed deep scanning method is a two-step searching approach that analyzes both time-domain signal energy and frequency-domain spectrum profiles. To start the detection process, a sample of the audio signal is first collected from the raw audio data. The sample data is cleaned and fed for detection by analyzing the time-domain signal energy and frequency-domain spectrum profile. The detection result is then recorded, and more data samples are collected for detection until all data are processed. Fig 4 shows the procedure of the detection process. The following three sections will introduce the cleaning, time-domain signal energy, and frequency-domain spectrum analysis.

### 2.5 Signal cleaning

**Fig 5:**
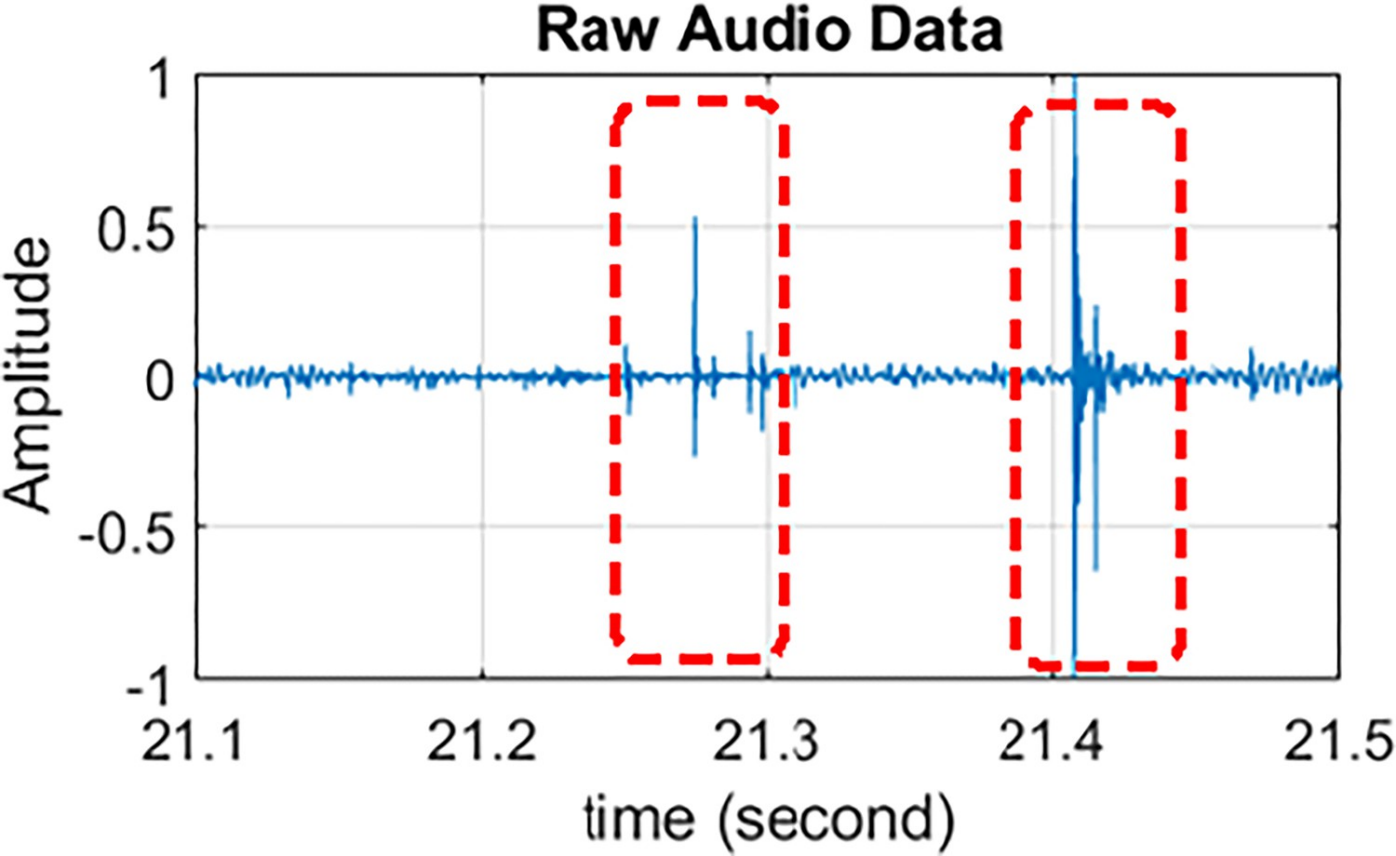
Snapping signals in raw audio data.

In order to obtain quality vessel signals for detection purposes, snapping shrimp snaps were removed from sound files. Fig 5 gives an example of two clusters of snap signals, one between 21.2 and 21.3 seconds and the other between 21.4 and 21.5 seconds. In the figure, the snap signals present amplitude spikes. Studies have shown that measured snap amplitudes generated by adult shrimp can reach above 190 dB 1μPa @ 1m, making snap signals one of the loudest sounds in the ocean [45]. In the spectrograms, the snapping sounds are presented by vertical stripes that usually cover the entire spectrum, even above 200 kHz [27].

**Fig 6:**
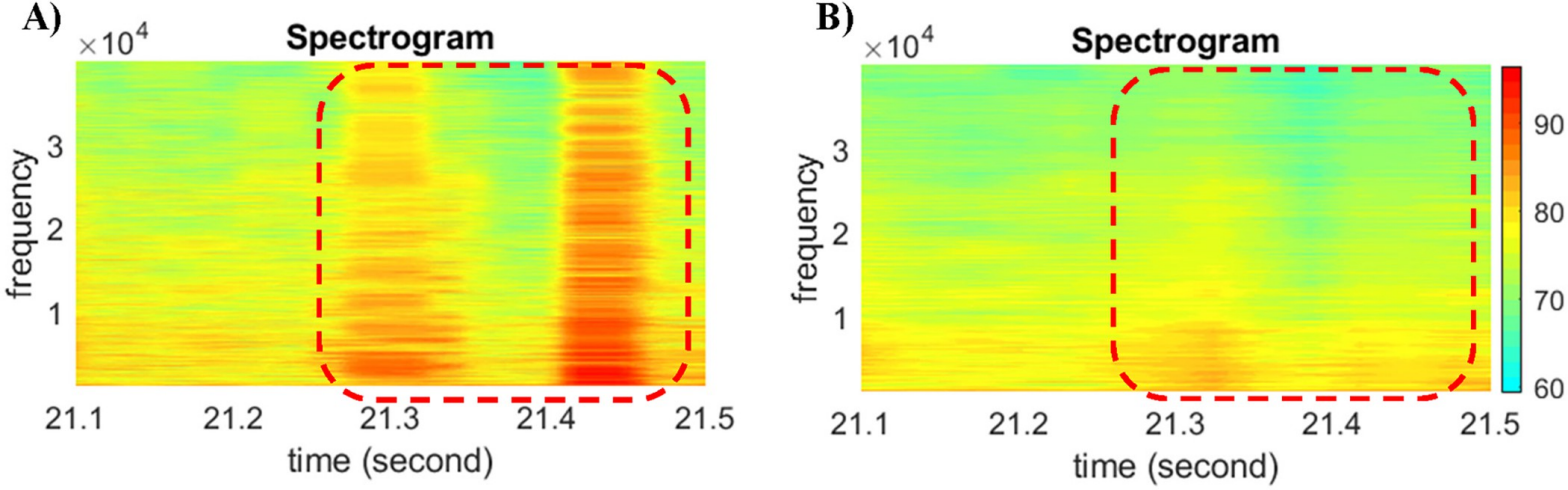
Signal cleaning examples removing snapping shrimp snaps. A) the original spectrogram diagram with snapping signals, B) the cleaned version of the spectrogram diagram. The color scheme in the spectrogram, changing from turquoise to light green, yellow, and red, symbolizes the increase of signal intensity (or loudness) of the signal over time at specified frequencies in the waveform.

Existing methods, such as averaging, filtering, and wavelet de-noising, may be adapted to reduce noise and thus clean snapping signals. This paper used a moving average method in the cleaning process. Fig 6 gives an example of the cleaning performance for signals introduced in Fig 5. Fig 6A represents the original signal in the spectrogram, and Fig 6B represents the cleaner version with snapping shrimp signals removed. The cleaned data is then used for the detection algorithm that will be introduced in Sections 2.6–2.7. It should be noted that while the cleaning process is not the focus of this study, in-depth research is necessary in the future.

### 2.6 Time-Domain signal energy

The purpose of the time-domain signal energy analysis is to quickly scan a.wav file to identify areas with high signal energy. These high-energy areas usually suggest active vessel activities and fish chorusing. To calculate the signal energy values, the raw audio data is first divided into multiple windows of data frames (e.g., 0.5 seconds per window), and each data frame is then transformed to discrete Fourier transformation (DFT) for signal energy computation. In this paper, we follow the methods proposed by Merchant et al. [48] and Monczak et al. [49], and use sound pressure level (SPL) to measure signal energy, as given in the formula:

$$SPL=10{log}_{10}\left(\frac{1}{p_{ref}^{2}}\sum_{f_{low}}^{f_{high}}\frac{P_{SS}(f)}{B}\right)-S$$ (1)

Where *p*_*ref*_ is a reference pressure of 1*μPa* for underwater measurements. *f*_*low*_ and *f*_*high*_ are lower and higher frequency values specifying the range of data signals for the SPL.

*P*_*ss*_(*f*) is the single-sided power spectrum, computed from the Discrete Fourier Transform (DFT) of the signal data sequence and divided by the length of the data.

*B* is the signal power bandwidth of the window function such as Hann window, which is used to offer some data overlap in time segments so that energy or spectral leakage could be restricted without spreading erroneously into other frequencies. For Hann window, B = 1.5 [48].

*S* is the correction factor that is determined by the hydrophone sensitivity or *M*_*h*_(*f*), system gain or *G*(*f*), and the zero-to-peak voltage, *V*_*ADC*_, of the analogue-to-digital converter:

$$S=M_{h}(f)+G(f)+20{log}_{10}(\frac{1}{V_{ADC}})$$ (2)

where hydrophone sensitivity = -185 dBV uPa^-1^; gain = 20 dB; and V_ADC_ = 1 volt.

**Fig 7:**
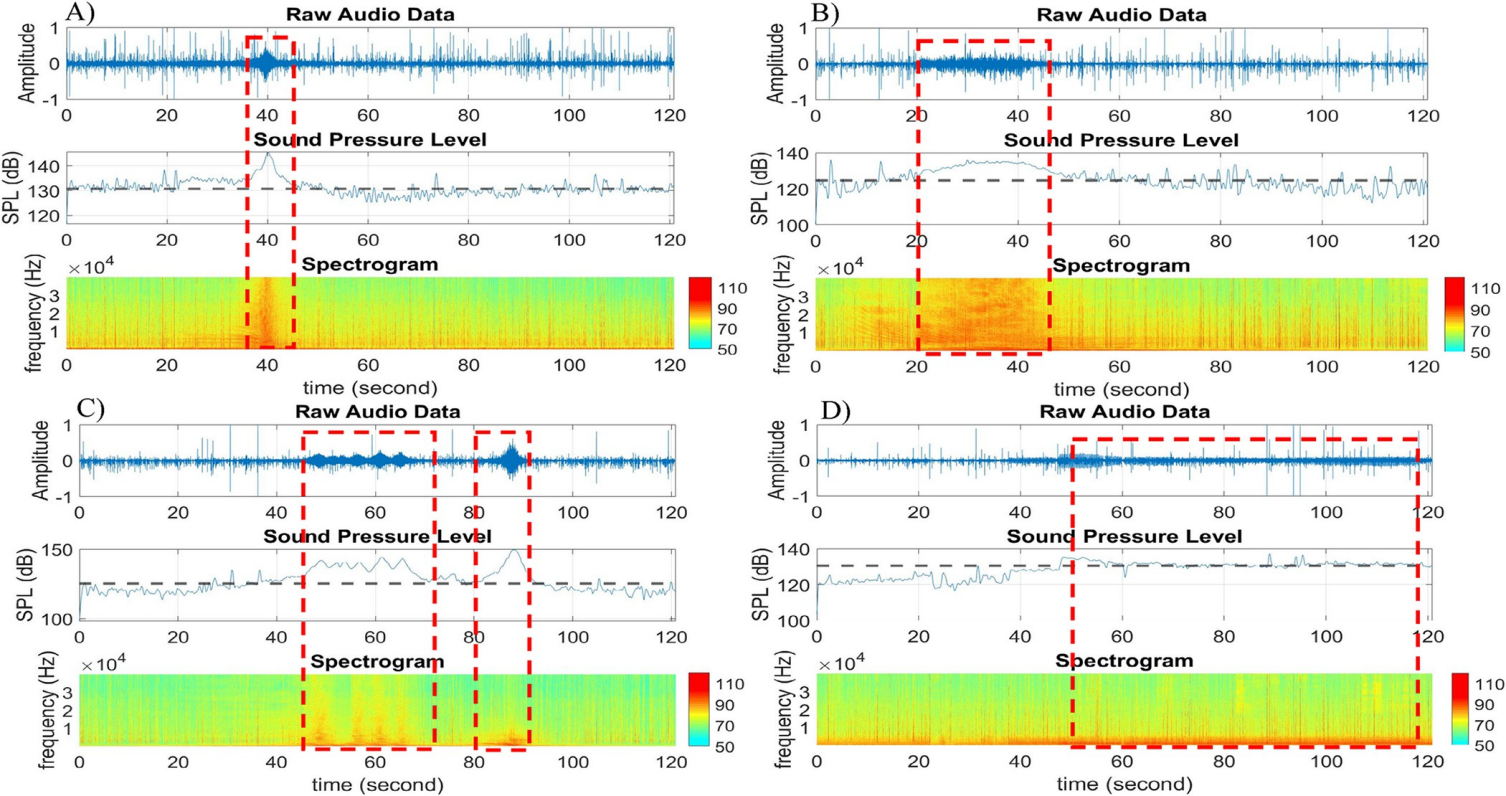
Examples of the time-domain signal energy analysis. A) Narrow burst broadband, B) wide burst broadband, C) narrow burst broadband (multiple signals), and D) loud signal without boat activity indicative of fish chorusing. Each panel includes a figure of raw audio data in the time domain (row 1), a figure of instantaneous broadband (1–40,000 Hz) SPL values (row 2), and a figure of the spectrogram of the corresponding audio data (row 3).

Fig 7 presents four example acoustic signals that may or may not include boat activities. Each example gives three diagrams, showing the raw audio data in the time domain (row 1), corresponding signal energy (i.e. SPL, broadband 1–40,000 Hz) values (row 2), and spectrogram of the audio data (row 3). The dotted line in the signal energy diagram (in row 2) represents the median value of the SPL values for the recorded acoustic data file. The median SPL values are computed for the entire 2-minute.wav file. If boat activity is present in the raw audio data, it triggers a higher level of energy. SPL values of audio signals with boat activity are much higher than the medium energy values (as highlighted in red dashed rectangles). This is consistent with all examples in Fig 7.

Fig 7D shows an example of audio data without boat activity, yet the energy diagram shows that a great portion of the audio signal presents at a higher energy than the median value. This finding indicates that higher SPLs do not necessarily suggest the existence of vessel noise and could indicate fish chorusing. This scenario necessitates an additional scanning process using the spectrogram.

### 2.7 Frequency-domain spectrum analysis

Following the previous step of the scanning process that analyzes the time-domain signal energy, a subset of data with higher levels of SPL values was identified. For the examples in Fig 7, the following sub-dataset might indicate the existence of vessel noise:

Fig 7A, sub-dataset between 35 seconds and 45 seconds

Fig 7B, sub-dataset between 20 seconds and 50 seconds

Fig 7C, sub-datasets between 40 seconds and 70 seconds, and between 80 seconds and 95 seconds

Fig 7D, sub-dataset between 40 seconds and 100 seconds

**Fig 8:**
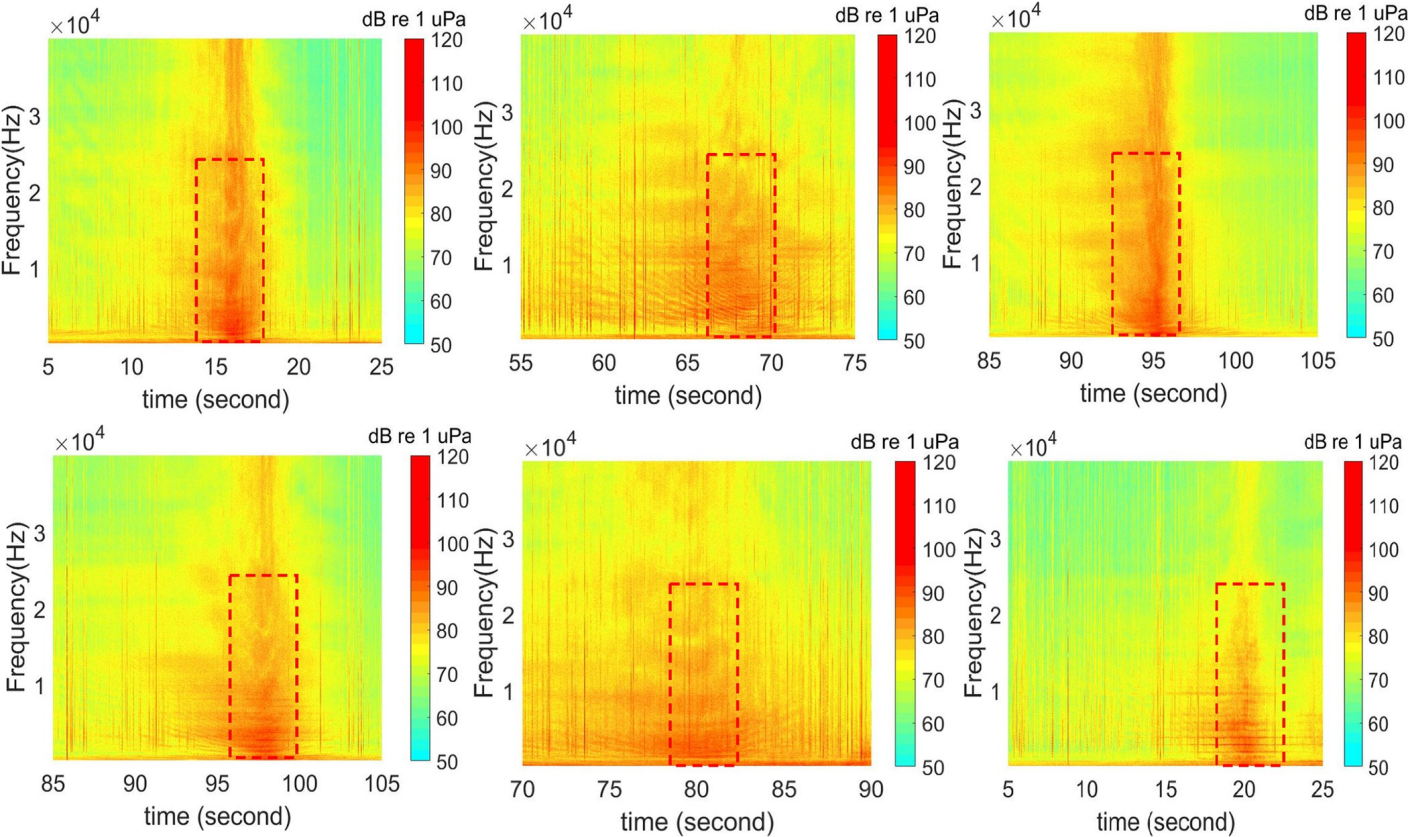
Possible boat signal features (raw data).

Using the frequency-domain spectrum profile (i.e., spectrogram image), many neural network techniques can be applied in the detection process. This concept process works similarly to handwritten character recognition using image detection. First, a set of features representing targeting objects (i.e., vessel signals) are collected. Fig 8 provides a set of six vessel signals. Each signal sequence is shown as a spectrogram with a 20-second window at the entire frequency range from 0 to 40 kHz. The selected feature could be characterized within a narrower time window, depending on the application needs, so that the feature would capture adequate details of the vessel signature but at the same time remain reasonably small in size. Hence, computing memory, data processing speed, and storage would not present a barrier for the application. Similarly, the selected feature may not need to cover the full frequency range. To demonstrate an example of the feature selection process, Fig 8 presents six feature options in dashed rectangles, where data approximately 5 seconds (Δt) in duration and 30 kHz (from 0 to 30 kHz) (Δf) are selected to represent a burst broadband, or BB, vessel signal.

**Fig 9:**
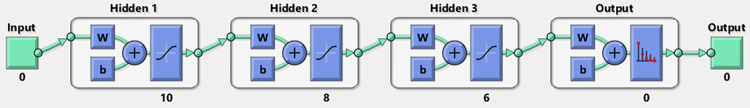
Example neural network.

To build a set of feature profiles {*Z*_*f*_}, an adequate number of features representing the targeted vessel signals must be collected. In addition, a set of regular audio data (without boat signals), {*Z*_*n*_}, must also be collected. Thus, a training dataset {*Z*} = {*Z*_*f*_, *Z*_*n*_} would be the input matrix used to feed to an applied neural network. Similarly, a target matrix {*T*} = {*T*_1_, *T*_0_} is used as the output matrix denoting the existence {*T*_1_} and non-existence {*T*_0_} of vessel noise, corresponding to the input matrix in the training dataset. When building a neural network, the number of hidden layers and corresponding neurons in each layer would also need to be specified. Fig 9 shows an example neural network using the MATLAB Deep Learning Toolbox. The example network has three hidden layers, and the size of the three layers are 10, 8, and 6 respectively; *w* and *b* indicate a weight matrix and a bias vector for the network.

### 2.8 Deep scanning algorithm

Accordingly, a complete deep scanning algorithm is given in Table 1.

**Table 1:** Deep scanning algorithm.

Algorithm: Deep Scanning Algorithm Pseudo code for processing]	
1: 2: 3: 4: 5: 6: 7: 8: 9: 10: 11: 12: 13: 14: 15: 16: 17: 18: 19: 20: 21: 22: 23: 24: 25: 26: 27: 28: 29: 30: 31: 32:	***[Build a neural network using feature training dataset]*** *Hidden_Layers = [10*, *8*, *6] [initialize the number of hidden layers and the size of each layer]* *net = build_net (Hidden_Layers)* *net_train = trainNetwork (net*, {*Z*_*f*_, *Z*_*n*_}, {*T*_1_, *T*_0_}*);* ***[Compute the time-domain signal energy]*** *SPL_Median = 0 [initial SPL median value]* *Mh = -185*.*5 [initialize hydrophone transducer sensitivity]* *G = 20 [initialize system gain]* *V_ADC = 1 [initialize zero-to-peak voltage of the analogue-to-digital converter]* *for a given frame of audio data y_s* *S = Mh +G+20log10(1/V_ADC)* Pss = 2*(DFT(yi) /size(y_s))^2 *SPL_s = 10log10(sum(Pss)/B)–S* *SPL_Median = median(SPL_s)* *end* ***[Deep Scanning Process]*** *for real-life audio data of y* *obtain a sample of data y_s to scan [a frame of audio data from the real-life audio data]* *compute instant SPL value (SPL(i)) and median SPL value (SPL_Median)* *for each instance y(i) with SPL(i) above the SPL_Median* *prepare spectrum data Y_i for detection* *detection = net{Y_i} [estimate the detection using the network]* *if successful [a positive estimate from the neural network]* *save result* *end* *end* *get more data samples* *end* *save data and display results*

### 2.9 Evaluation settings and reporting effectiveness of neural network detector

To prepare targeted vessel feature profiles of {*Z*_*f*_}, this research focused on BB signals only (i.e., Burst Narrow and Burst Wide), which is a limitation in automated detection of vessel noise because many vessels produce VB and LF signals depending upon speed and vessel type. However, VB and LF vessel signatures are more challenging to distinguish, especially LF vessel noise because of their similarity to fish choruses. In addition, spectrogram features were selected in a window that spanned from 800 to 10,000 Hz in frequency (Δ*f*) and lasted about 5.0 to 10.0 seconds in time (Δ*t*, centered at each instance). The frequency above 800 Hz was selected because, in the spring and summer, lower frequency bandwidths (e.g., 50–800 Hz) usually include fish calling and chorusing (Fig 3A). Data in higher frequency ranges (i.e., 10 to 40 kHz) might be valuable, but it significantly increases data storage. Thus, it may be considered in future studies.

Based on the average burst duration introduced in Section 2.3, this research studied 10 sets of feature profiles, using Δt between 5.0 and 7.5 seconds, to evaluate the performance of the introduced method. Each feature set {*Z*} included about 350 to 850 classifiers selected from stations 14M (i.e., 2017 and 2018) and 37M (i.e., 2018). Because of the relatively small feature dataset, a three-layer neural network using the MATLAB Deep Learning Toolbox was constructed for this study. The neural network was then trained in each of the feature set {*Z*}, then the trained network was applied to all raw audio data for detection. Neural network detections of BB signals were compared to manually observed detections. As mentioned previously, the manual examination involved individual verification of each audio file, using Adobe Audition, to visually scan and listen to two-minute.wav files to validate the existence of BB vessel noise.

The evaluation was performed in each.wav file using the algorithm given in Section 4.4, and an error was marked for the file if either 1) the algorithm detected a BB vessel signal but the file did not contain one or 2) the algorithm did not detect a BB signal but the file did contain one. If a dataset of *N* total.wav files had *n*_*ε*_ error detections, the accuracy of the detection *ρ* was determined by:

$$\rho =\frac{N-n_{\varepsilon }}{N}$$ (3)

## 3. Results

### 3.1 Neural network detections compared to manual review analysis

Table 2 below gives the detection results using two representative feature profiles, V_38 and V_389A, both using a feature of Δt = 5.5 seconds and Δf between 800 and 10,000 Hz. V_38 includes a total of 4274 feature profiles formed by 259 burst signals (Burst Narrow and Burst Wide) as well as 4015 non-burst signals. V_389A includes all feature signals from V_38 but with additional features with the purpose of performance improvement. In total, V_389A contains 5035 feature profiles, including 317 burst signals and 4718 non-burst signals.

**Table 2:** Detection accuracy.

Year	Station	Dataset	# of.wav Files Processed	Detection accuracy *ρ*	
				V_38_SPL	V_389A_SPL
2017					
	37M	1216_020217	4188	99.21%	96.99%
		1217_050317	1371	97.67%	97.23%
		1216_072817	6184	97.70%	97.95%
		1217_102417	6346	96.74%	97.08%
	14M	1215_020217	6192	97.75%	97.81%
		1154_050317	6481	96.79%	96.93%
		1215_072817	6186	*93*.*52%*	*93*.*60%*
		1216_102417	6419	*94*.*72% *	*94*.*67% *
	9M	1082_020217	6126	99.82%	99.80%
		1153_050317	6502	99.77%	99.80%
		1082_072817	6189	98.09%	98.01%
		1153_102417	6424	98.55%	98.63%
2018					
	37M	1216_012318	6634	99.29%	99.35%
		1217_042018	6274	99.17%	99.11%
		1084_072618	6815	*94*.*69%*	*94*.*63%*
		1217_101618	5909	97.29%	97.66%
	14M	1215_012318	6635	99.38%	99.34%
		1154_042018	6273	99.27%	99.40%
		1084_072618	6815	97.81%	97.78%
		1154_101618	5912	98.36%	98.73%
	9M	1082_012318	6635	99.83%	99.74%
		1153_042018	6274	99.86%	99.90%
		1082_072618	6815	99.71%	99.77%
		1153_101618	5914	98.81%	98.83%
2021					
	37M	1216_012521	2158	99.60%	98.40%
		1152_042621	2186	99.70%	99.10%
		1216_072621	2191	99.40%	98.70%
		1152_101921	2043	99.30%	99.20%
	14M	1215_012521	2159	99.80%	99.63%
		1084_042621	2189	99.95%	99.63%
		1215_072621	2192	99.45%	99.31%
		1084_101921	2044	99.31%	99.31%
	9M	1083_012521	2160	99.91%	99.91%
		1081_042621	2189	99.95%	99.91%
		1081_072621	2193	99.82%	99.77%
		1081_101921	2045	99.95%	99.66%
**Overall Average:**			**171,262 Total.wav Files**	**99.15%**	**99.03%**

### 3.2 Sound pressure levels and sound exposure levels of vessel noise

**Fig 10:**
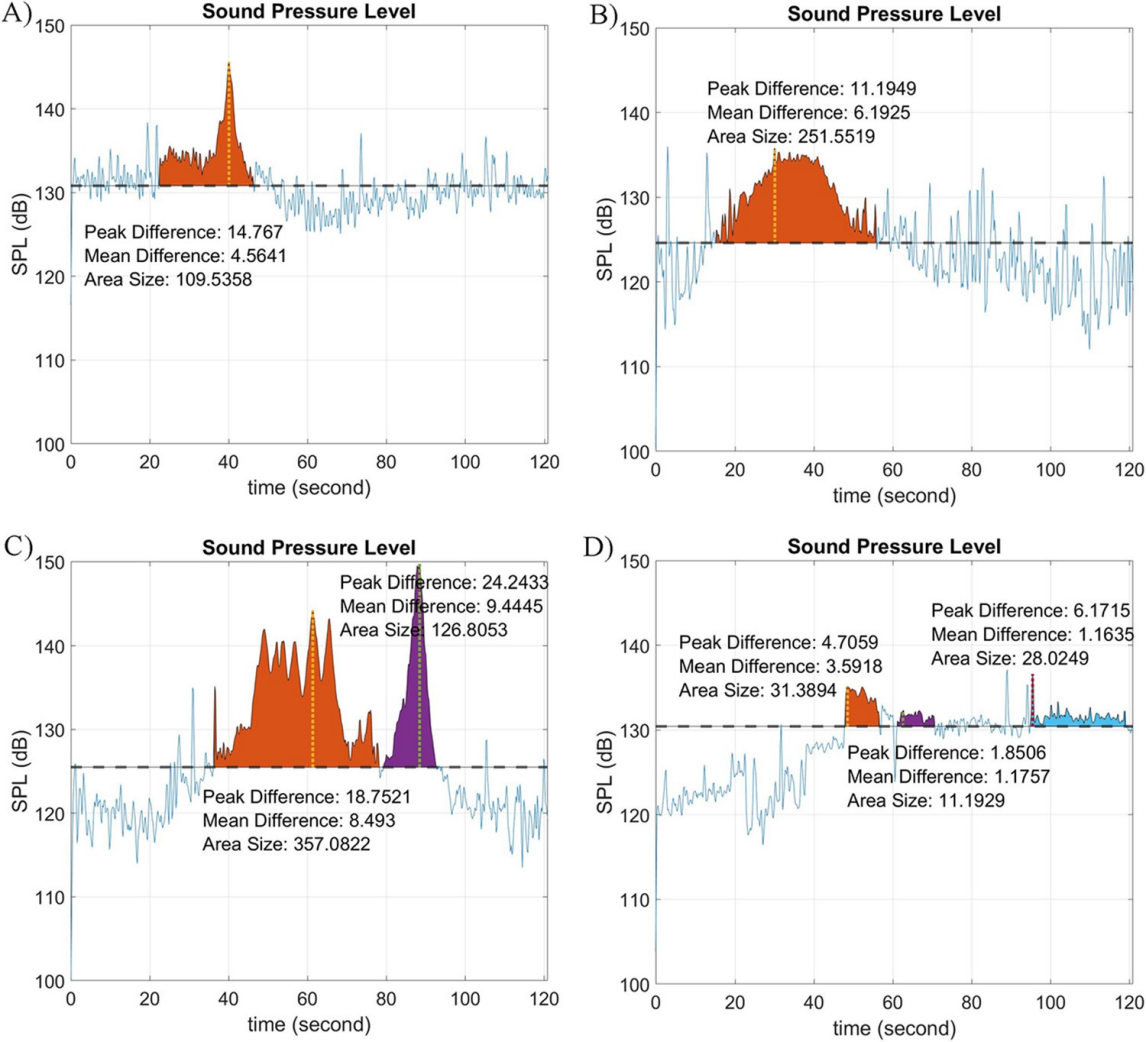
Sound pressure level diagram with peak difference, mean difference, and area size for vessel detection. The dotted line represents the median SPL of the entire two-minute.wav file. A) SPL values for a narrow-burst signal in Fig 7A, 7B) SPL values for a wide burst signal in Fig 7B, 7C) SPL values for a multiple narrow bursts signal in Fig 7C, and 7D) SPL values for a signal without vessel noise in Fig 7D. The Area Size is equal to the sound exposure level (SEL) for each vessel detection. In the case of Fig 7D, this sound is most likely a fish chorus and would not be included in cumulative noise energy (SEL_cum_) associated with vessels.

Section II.F “Time-Domain Signal Energy” used four examples (Fig 7A–7D) to showcase the potential of using SPL values to identify vessel noise. A closer inspection of the signal energy (i.e., SPL values) would be valuable for future research, especially in automating sound exposure levels (SEL) from each vessel detected. Sound exposure level takes into account the received level and duration of vessel noise. Fig 10 below exploited SPL values of all four examples in Fig 7, where a peak difference, a mean difference, and the SPL area are presented. The Peak Difference gives the SPL difference between the peak and median values; the Mean Difference gives the SPL difference between the average (of signal values above the median line) and the median values; and the Area Size refers to the sum of trapezoidal areas between the SPL curve and the median line. The respective Area Size is equal to the SEL for each vessel (or other significant sound sources) detected. By integrating all vessel SELs, one can derive a cumulative noise energy (SELcum) for each station. Cumulative noise energies can then be compared among estuaries (e.g., an urbanized port like Charleston Harbor versus a less impacted estuary like the May River) to better understand the impacts of noise on marine life.

### 3.3 Long-term time series of vessel noise

**Fig 11:**
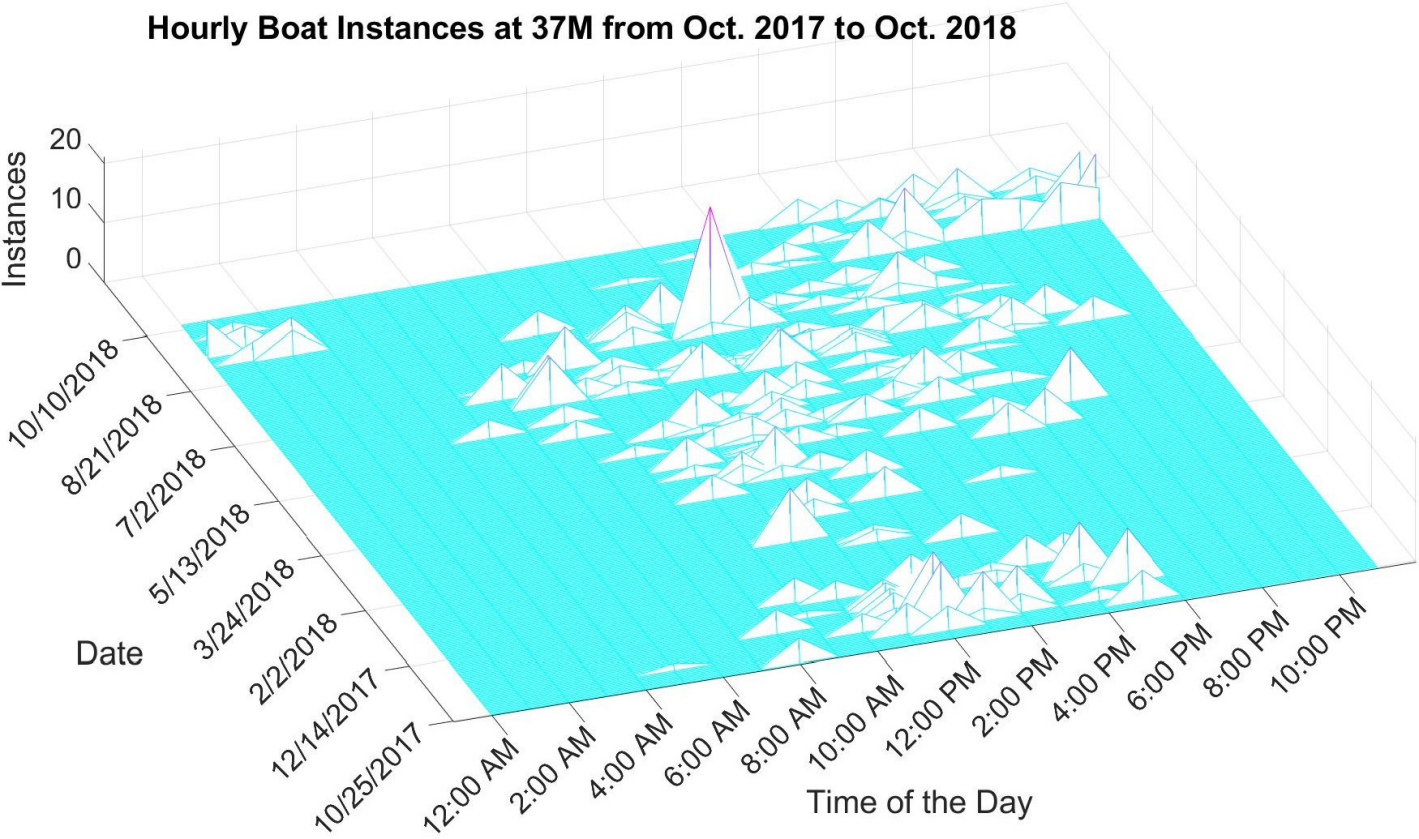
Time series of vessel noise detections at station 37M in the May River, SC.

Fig 11 provides a time series (i.e., October 2017 –October 2018) of vessel noise detections at station 37M in the May River acquired using the deep scanning algorithm. The x-axis represents the time of the day, the y-axis is the date, and the z-axis is the number of vessel detections. Vessel noise is detected more frequently during the day. More vessel detections occur in the summer as compared to the winter, illustrating the increased use of recreational vessels during the warmer months. It is interesting to see some boat activities occurring in the late evening between late September and October of 2018. A close investigation revealed that, due to Hurricane Michael (Oct 10, 2018), boats and ships from nearby ports such as Charleston were relocated to the May River estuary prior to the hurricane, in addition, night dredging activities might also have been conducted to clear the waterway. These boat activities were recorded in the DSG-Oceans acoustic recorder at 37M and thus reflected in the diagram. It should also be noted that due to the low recording frequency, i.e., 2 minutes of recording every 20 minutes, the data presented in the diagram may only reflect a very small portion of the real vessel activities, however, the limited data does provide valuable information for many applications.

## 4. Discussion

### 4.1 Performance analysis

The proposed Deep Scanning method proved valuable. Both feature profiles, V_38_SPL and V_389A_SPL, offer very similar results in each dataset at all stations across all years. In addition, the overall average accuracy results are similar. It should be noted that results from 14M in 2017 (1215_072817, 1217_102417) and 37M in 2018 (1084_072618) are less effective than other datasets, at about 94%. When comparing the detection performance among stations, it appears that the performance increases from 37M to 14M to 9M. One explanation is that the tidal river estuary increases in width from 9M to 37M, so vessels travel closer to recording stations near the headwaters (i.e., 9M and 14M) as compared to the mouth (i.e., 37M). Thus, the feature profiles of vessel noise occurring in the headwaters are less diverse and the number of classifiers created was sufficient to characterize vessel noise in this area of the estuary. However, at 37M near the intra-coastal waterway, the feature profiles of vessel noise occurring at the mouth are more abundant and diverse, and the number of classifiers created was insufficient to characterize vessel noise. Another explanation is that the soundscape is generally more complicated at station 37M. Research has shown that diverse sound-producing species, including snapping shrimp, silver perch, oyster toadfish, black drum, spotted seatrout, and red drum, as well as various human activities, are actively contributing to the underwater soundscape near station 37M [45] [49, 50]. In contrast, acoustic activity is less diverse near the headwaters (i.e., station 9M).

### 4.2 Future improvements

The evaluation adopted a simple 3-layer neural network in order to minimize computational resources and processing time. In the future, the training dataset could be larger; therefore, the neural network could be deeper (with more hidden layers), so that better learning performance and detection results are achieved.

The goal of a comprehensive detection algorithm for vessel noise in estuaries of the southeast USA is to distinguish all vessel noise even in the presence of fish chorusing, which is a dominant feature in estuarine soundscapes. In the future, the neural network algorithm should also include classifiers for VB and LF vessel noise signatures. This approach would provide a comprehensive detection process for all vessels in an estuary. Additionally, sound exposure levels (SEL) from each vessel detection could be integrated into the detection algorithm. Sound exposure level takes into account the received level and duration of vessel noise. By integrating all vessel SELs, one can derive a cumulative noise energy (SELcum) for each station. Cumulative noise energies can then be compared among estuaries (e.g., an urbanized port like Charleston Harbor versus a less impacted estuary like the May River) to better understand the impacts of noise on marine life.

## 5. Conclusions

This research presents a deep-learning, detection method, called Deep-Scanning, to identify vessel signals from underwater acoustic measurements. The focus is on the application of existing deep-learning tools to identify vessel noise so that approaches can be developed to identify other sound-producing organisms in the future.

The Deep-Scanning method involves a time-domain noise energy and frequency-domain stage. The stage of the time-domain noise energy analysis identifies possible vessel signals based on signal energy levels measured from SPLs. Then, the stage of the frequency-domain spectrum analysis builds a neural network to examine the focused signals and detect vessels. Using audio files from an Estuarine Soundscape Observatory Network in the Southeast (ESONS), the research first built a set of classifiers with a combination of different time durations and frequency bands. A simple 3-layer neural network was then constructed to train each of the feature datasets. The network was then used to evaluate the detection method using real measurement data collected in the years 2017, 2018, and 2021 over three locations in the May River, SC. Human observers scanned 171,262 audio files for the presence of vessel noise, and these detections were compared to the Deep-Scanning approach. The neural network for BB signals achieved an average accuracy of 99.0%. With the automatic detection method, a time series of vessel detections near a deployment station was also presented for visualization.

## Supporting information

S1 Data (ZIP)
